# Myocardial perfusion and cardiac dimensions during extracorporeal membrane oxygenation–supported circulation in a porcine model of critical post-cardiotomy failure

**DOI:** 10.1177/0267659120907557

**Published:** 2020-03-04

**Authors:** Atle Solholm, Pirjo-Riitta Salminen, Lodve Stangeland, Christian Arvei Moen, Arve Mongstad, Bård Svenheim, Rune Haaverstad, Ketil Grong

**Affiliations:** 1 Section of Cardiothoracic Surgery, Department of Heart Disease, Haukeland University Hospital, Bergen, Norway; 2 Department of Clinical Science, Faculty of Medicine, University of Bergen, Bergen, Norway

**Keywords:** venoarterial extracorporeal membrane oxygenation, post-cardiotomy failure, haemodynamics, myocardial perfusion, left ventricle dimension

## Abstract

**Introduction:**

Venoarterial extracorporeal membrane oxygenation is widely used as mechanical circulatory support for severe heart failure. A major concern with this treatment modality is left ventricular distension due to inability to overcome the afterload created by the extracorporeal membrane oxygenation circuit. The present porcine study evaluates coronary circulation, myocardial perfusion and ventricular distension during venoarterial extracorporeal membrane oxygenation.

**Methods:**

Ten anesthetized open-chest pigs were cannulated and put on cardiopulmonary bypass. Heart failure was achieved by 90 minutes of aortic cross-clamping with insufficient cardioplegic protection. After declamping, the animals were supported by venoarterial extracorporeal membrane oxygenation for 3 hours. Continuous haemodynamic measurements were performed at baseline, during cardiopulmonary bypass/aortic cross-clamping and during venoarterial extracorporeal membrane oxygenation. Fluorescent microsphere injections at baseline and after 1, 2 and 3 hours on venoarterial extracorporeal membrane oxygenation evaluated myocardial perfusion. Left ventricular function and distension were assessed by epicardial echocardiography.

**Results:**

The myocardial injury caused by 90 minutes of ischaemia resulted in a poorly contracting myocardium, necessitating venoarterial extracorporeal membrane oxygenation in all animals. The circulatory support maintained the mean arterial blood pressure within a satisfactory range. A hyperaemic left anterior descending coronary artery flow while on extracorporeal membrane oxygenation was observed compared to baseline. Myocardial tissue perfusion measured by microspheres was low, especially in the subendocardium. Echocardiography revealed myocardial tissue oedema, a virtually empty left ventricle, and a left ventricular output that remained negligible throughout the extracorporeal membrane oxygenation run.

**Conclusion:**

Coronary artery blood flow is maintained during venoarterial extracorporeal membrane oxygenation after cardiopulmonary bypass and cardioplegic arrest despite severely affected performance of the left ventricle. Myocardial perfusion decreases, however, presumably due to rapid development of myocardial tissue oedema.

## Introduction

Venoarterial extracorporeal membrane oxygenation (VA-ECMO) has become an important treatment modality in patients with cardiogenic shock.^1^ In cardiac surgery, VA-ECMO is used as temporary circulatory support for days or weeks if weaning from the heart–lung machine is problematic.^2^ VA-ECMO has the potential of full circulatory support for the patient, preserving all vital organ functions.^3^

A major concern with VA-ECMO as circulatory support is left ventricular distension due to the inability of the failing heart to generate sufficient ejection pressure and empty into the ascending aorta against the afterload (i.e. the aortic pressure) generated by the ECMO unit. Failure to decompress the left ventricle (LV) might delay myocardial restitution.^4,5^ In the clinical setting, this is seen as a more or less flat arterial pressure curve in a patient with spontaneous cardiac activity on VA-ECMO. Echocardiographic evaluation would reveal left ventricular distension, indicating high intraventricular pressures and compromised myocardial perfusion.^6,7^ The aim of the present study is to evaluate an animal model for critical post-cardiotomy failure and VA-ECMO-supported circulation, focusing on the relationship between left ventricular distension, coronary circulation and myocardial perfusion early after aortic declamping.

## Methods

The experimental protocol was ethically evaluated and approved by the Norwegian State Commission for Laboratory Animals (Project: 201710294), and performed in accordance with the European Communities Council Directive of 2010 (63/EU). Ten young pigs (NOROC) of either gender, weighing 46 ± 5 kg (standard deviation (SD)) were used. The animals were premedicated with 20-mg/kg i.m. ketamine, 10-mg diazepam and 1-mg atropine, and ventilated on mask with oxygen with 3% isoflurane (Rhodia, Bristol, United Kingdom) for a short time to establish intravenous (IV) access through two ear veins. After inducing anaesthesia with loading doses of fentanyl (0.02 mg/kg), midazolam (0.3 mg/kg), pancuronium (0.063 mg/kg) and pentobarbital (15 mg/kg), the animals were tracheotomised, intubated and ventilated (Julian, Drägerwerk, Lübeck, Germany) with nitrous oxide (57-58%) and oxygen. Continuous IV infusions of fentanyl (0.02 mg/kg/h), midazolam (0.3 mg/kg/h), pancuronium (0.2 mg/kg/h) and pentobarbital (4 mg/kg/h) maintained anaesthesia. The pentobarbital infusion constituted the basic fluid substitution, 15 mL/kg/h of Ringer’s acetate. This anaesthetic protocol has previously been thoroughly evaluated, justifying safe use of neuromuscular blocking agents in young pigs.^8^

### Instrumentation and evaluation

A urinary catheter was introduced through an open suprapubic cystotomy. Rectal temperature was monitored, and a pulse oximeter was applied to the tail. The iliac vessels were accessed through inguinal incisions, and the right carotid artery through the tracheostomy incision. A 6-Fr radial introducer (Radifocus Introducer II, Terumo Europe, Leuven, Belgium) was introduced in the right carotid artery, another in the right external iliac artery and a third one in the left external iliac vein. Heparin 125 IU/kg was administered IV to prevent catheters clotting. A microtip pressure catheter (MPC-500, Millar Instruments, Houston, TX, United States) was inserted into the proximal aorta through the right carotid artery.

A median sternotomy and pericardiotomy were performed. A continuous cardiac output catheter (CCO/EDV 177 HF 75, Edwards Lifesciences Inc., Irwin, CA, United States) was introduced through the left internal mammary vein and floated into the pulmonary artery, monitoring CCO, central venous pressure (CVP) and pulmonary artery pressure (PAP) (Vigilance II^®^ and TruWave^®^, Edwards Life Sciences Inc.). A second microtip pressure catheter (MPC-500, Millar Instruments) was inserted into the LV from the apex of the heart. An infant feeding tube was inserted into the left atrium via the auricle for injection of microspheres. Blood flow rate in the proximal left anterior descending coronary artery (LAD) was measured with a transit-time flowmeter (CM4000, Medistim, Oslo, Norway). Haemodynamic variables were continuously recorded, digitized and later analysed (ACQ-7700 and Physiology Platform v.5.20, Data Sciences International, St. Paul, MN, United States).

After administration of systemic Heparin 250 IU/kg, the right external iliac vein and the left external iliac artery were cannulated with heparin-coated 23 Fr venous and 17 Fr arterial ECMO cannulas (HLS™, Maquet Cardiopulmonary GmbH, Rastatt, Germany). The heparin-coated ECMO circuit (PLS Advanced 5.0, Maquet Cardiopulmonary GmbH) was primed with Ringer’s acetate. The centrifugal pump was driven by a Maquet Rotaflow ECMO unit (Maquet Cardiopulmonary GmbH). A repeated dose of Heparin 125 IU/kg was given after 45 minutes of aortic cross-clamping.

### Experimental protocol

After instrumentation baseline variables were registered, including haemodynamic variables, arterial blood gases (OPTI CCA-TS2, OPTI Medical Systems, Atlanta, GA, United States) and the first injection of 15-μm fluorescent microspheres (Dye-Trak ‘F’; Triton Technology Inc., San Diego, CA, United States). After pre-oxygenation (100% O_2_ on the respirator) and during brief periods of respiratory shut-off, echocardiographic recordings in end-expirium were obtained. In the four chamber long-axis view, the left ventricular end-diastolic and end-systolic septal to free wall and apex to basis diameters were measured with echocardiography (Vivid E9, GE Vingmed Ultrasound, Horten, Norway). The corresponding anterior–posterior and the septal to free wall diameters were measured from the short-axis view, together with the anterior wall thickness. End-diastole was defined as the first deflection of the QRS-complex in the electrocardiogram (ECG), start and end of ejection were defined as the opening and closure of the aortic valve by pulsed wave Doppler (PWD). Recordings were analysed with a commercial software (EchoPAC BT12, GE Vingmed Ultrasound).

During cardioplegic arrest, the ECMO unit was used for cardiopulmonary bypass (CPB) with a pump flow set to 90 mL/kg/min. The aorta was cross-clamped. Lukewarm crystalloid cardioplegia (St. Thomas’ Hospital No. 2) was infused in the aortic root and immediately discontinued once ECG was isoelectric. Ventilation was reduced to half of the original setting during this period of CPB. The aortic cross-clamp was maintained for 90 minutes, and then released for the heart to be reperfused. Any ventricular fibrillation was immediately electroconverted. Once a spontaneous cardiac rhythm was regained, within minutes after declamping, flow on the ECMO unit was decreased to 72 mL/kg/min, balancing between suck-down and fluid overload. Whenever heart rate exceeded 200 beats/min, synchronized electroconversion was performed, and if the heart rate was below 100 beats/min, a pacemaker set at 100 in ‘on-demand’ mode was allowed to pace the heart by use of epicardial electrodes on the right ventricle. After aortic declamping, an additional infusion of Ringer’s acetate was commenced with 10 mL/kg/h. At any sign of imminent suck-down, bolus doses of 60-mL Ringer’s acetate were given until the problem ceased. Arterial blood gases, microsphere injections into the ECMO arterial line and echocardiographic recordings were repeated at 60, 120 and 180 minutes on ECMO-supported circulation.

### Myocardial and renal tissue samples

After euthanasia (intracardiac potassium chloride), tissue samples were obtained from the endo-, mid- and epicardium of the anterior, posterior and the septal wall of the LV, from the right ventricle and from both kidneys. The samples along with reference blood samples were weighted and hydrolysed, with subsequent isolation of microspheres by filtration, dissolving of the fluorescent colours and quantification with fluorospectrophotometry (RF-5301PC; Shimadzu, Kyoto, Japan).^9^ Separate tissue samples were weighed, dried at 60°C for 3 weeks, reweighed and water content calculated as fraction of wet weight.

### Statistical analysis

Data were analysed using SPSS v. 23 (IBM Corp., Armonk, NY, United States). Baseline values are given as mean ± SEM (standard error of the mean) or median (first quartile; third quartile) for variables with normal or skewed distribution. Haemodynamic variables were evaluated by one-way analysis of variance for repeated measurements (RM-ANOVA) or by Friedman Repeated Measures Analysis of Variance on Ranks whenever appropriate. If significant, time trend analysis using linear regression analysis with replication and testing for linearity was performed. Left ventricular tissue blood flow rates during ECMO-assisted circulation was evaluated by two-way analysis of variance (ANOVA) with time and wall layer within the same heart as repeated/related factors. If the interaction between time and layer were significant (p < 0.10), new ANOVAs for simple main effects were performed followed by Holm–Sidak multiple contrast tests when appropriate. Except for the interaction effect, a p-value <0.05 was regarded as significant.

## Results

### Characteristics at baseline and during CPB with aortic cross-clamp

The haemodynamic variables at baseline are presented in Table 1. Arterial blood gases and acid–base status were within physiological limits (Supplemental Table A).

**Table 1.** Haemodynamic variables in the baseline situation.

Heart rate (beats/min)	91 ± 4
LVSP_max_ (mmHg)	103 (100; 107)
LVEDP (mmHg)	8.6 ± 0.5
LV-dP/dt_max_ (mmHg/s)	1,256 ± 54
LV-dP/dt_min_ (mmHg/s)	−1,828 ± 63
CO (L/min)	4.0 ± 0.2
MAP (mmHg)	87 ± 3
CVP_mean_ (mmHg)*	7.7 ± 0.9
PAP_mean_ (mmHg)	19 ± 1

LVSP_max_: peak systolic left ventricular pressure; LVEDP: left ventricular end-diastolic pressure; LV-dP/dt_max_ and LV-dP/dt_min_: peak positive and peak negative of first derivative of left ventricular pressure; CO: cardiac output; MAP: mean aortic pressure; CVP_mean_: mean central venous pressure; PAP_mean_: mean pulmonary artery pressure.

Values are mean ± SEM or median (first quartile; third quartile) for 10 anaesthetized pigs.

* n = 9 due to technical failure.

**Figure 1.**
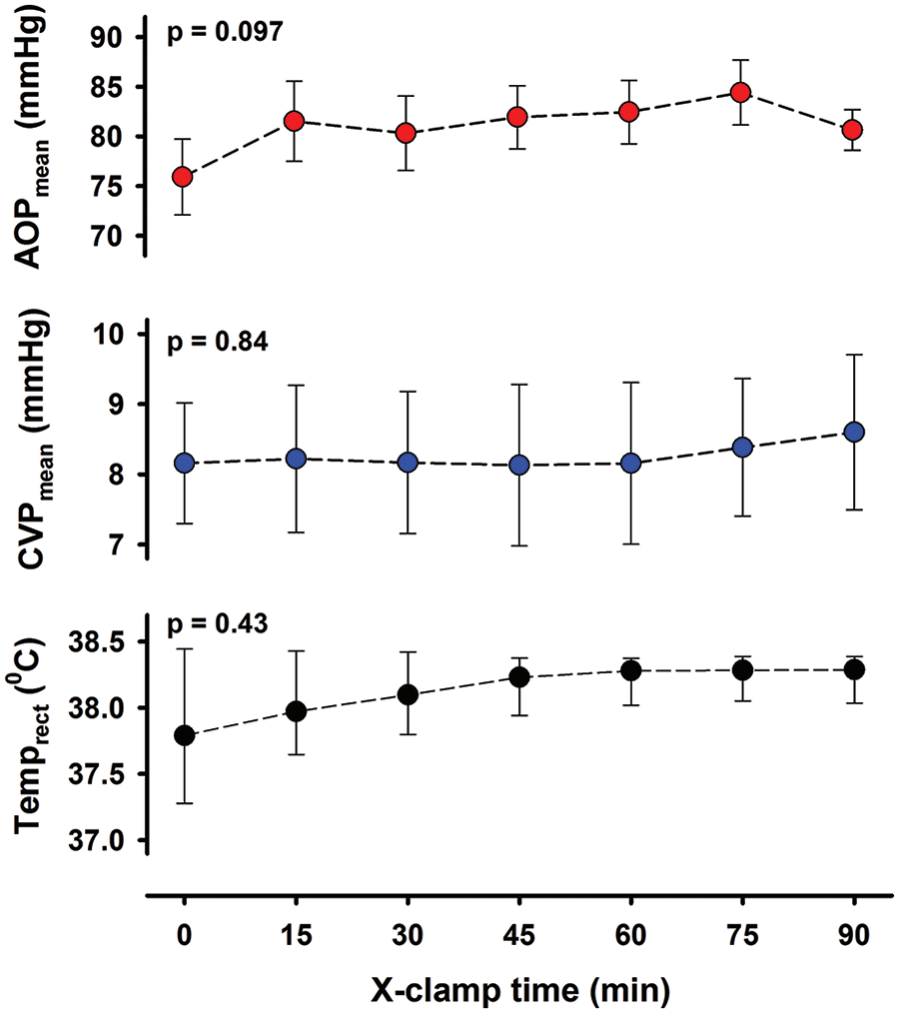
Variables during 90 minutes of aortic cross-clamping and cardioplegic arrest. AOP: aortic pressure; CVP: central venous pressure; Temp_rect_: rectal temperature. Variables are mean ± SEM or median with first and third quartiles, n = 10 (9 for CVP). p is the probabilities by one-way ANOVA for repeated measurements or Friedman Repeated Measures ANOVA on ranks.

An average of 266 mL (range: 95-522 mL) of antegrade tepid crystalloid cardioplegia was used to obtain isoelectric ECG. During aortic cross-clamping, mean aortic blood pressure was slightly lower than at baseline, but stable (Figure 1). Both CVP and body temperature remained within normal range. Arterial blood gas parameters were stable during CPB and cross-clamp (Supplemental Table A).

### Haemodynamic variables on ECMO

**Figure 2.**
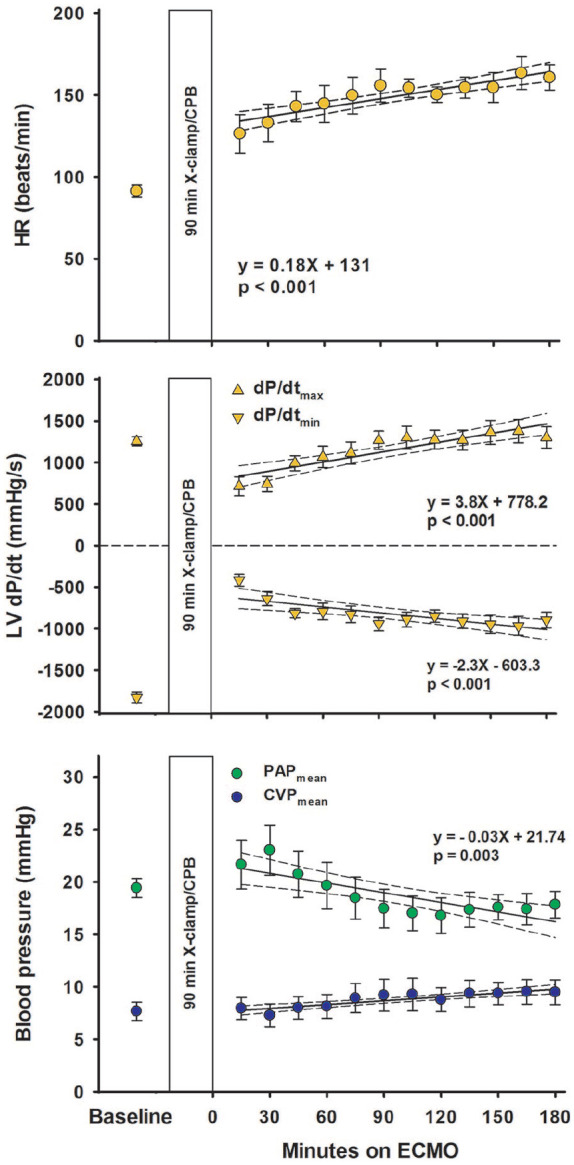
Haemodynamic variables at baseline and on ECMO-supported circulation following 90 minutes of aortic clamping and cardioplegic arrest. HR: heart rate; LV dP/dt: first derivative of left ventricular pressure. Mean pulmonary artery pressure (PAP; n = 10) and central venous pressure (CVP; n = 9) values are mean ± SEM. Regression lines with 95% confidence limits.

**Figure 3.**
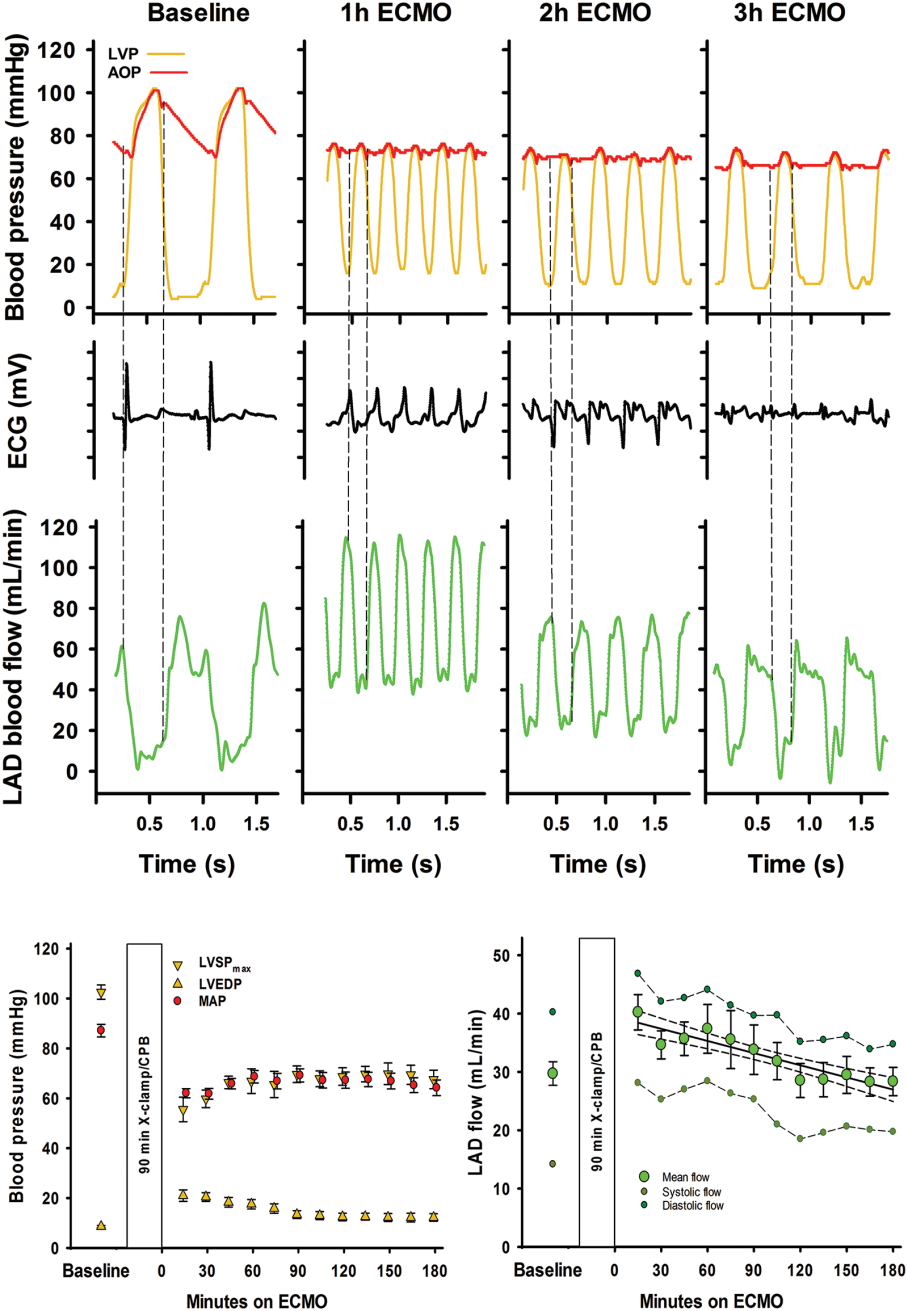
Recordings from a typical experiment at baseline and 1, 2 and 3 hours on ECMO-supported circulation after cardioplegic arrest and time plots for blood pressures and coronary blood flow. LVP and AOP: left ventricular and central aortic pressures; LAD: left anterior descending coronary artery; LVSP_max_ and LVEDP: left ventricular peak systolic and end-diastolic pressures; MAP: mean aortic pressure. Values are mean ± SEM (n = 10). Regression line with 95% confidence limits.

After removal of aortic cross-clamp, electroconversion of ventricular fibrillation and ECMO-supported circulation was necessary in all animals. Heart rate and LV-dP/dt_max_ gradually increased over time whereas LV-dP/dt_min_ became more negative (Figure 2), mean PAP gradually decreased and CVP was unchanged. With ECMO-supported circulation, the mean aortic blood pressure averaged 62 ± 2 mmHg 15 minutes after aortic declamping and was essentially unchanged during the 180 minutes of observation (Figure 3). The systolic–diastolic pulsatility in the aorta averaged between 4.5 ± 0.9 and 6.9 ± 1.0 mmHg during the 3 hours on ECMO. The left ventricular pressure curve demonstrated the typical systolic-to-diastolic wave pattern throughout the ECMO-supported period. Whereas LVSP_max_ gradually increased with time from 56 ± 5 mmHg after 15 minutes on ECMO to 68 ± 4 mmHg after 180 minutes (p = 0.005), left ventricular end-diastolic pressure (LVEDP) decreased from 21.0 ± 2.3 to 12.2 ± 1.5 mmHg (p < 0.001).

### Coronary blood flow and myocardial perfusion

A hyperaemic response in the proximal LAD flow was observed following aortic declamping. After 15 minutes of reperfusion, LAD blood flow rate was on average increased by approximately 50% compared to baseline. LAD flow rate decreased over time levelling off at baseline levels at 120 minutes on ECMO-supported circulation (Figure 3). Throughout the 180-minute ECMO period, there was a typical coronary artery flow pattern in the LAD with high diastolic- compared to systolic blood flow rate.

**Figure 4.**
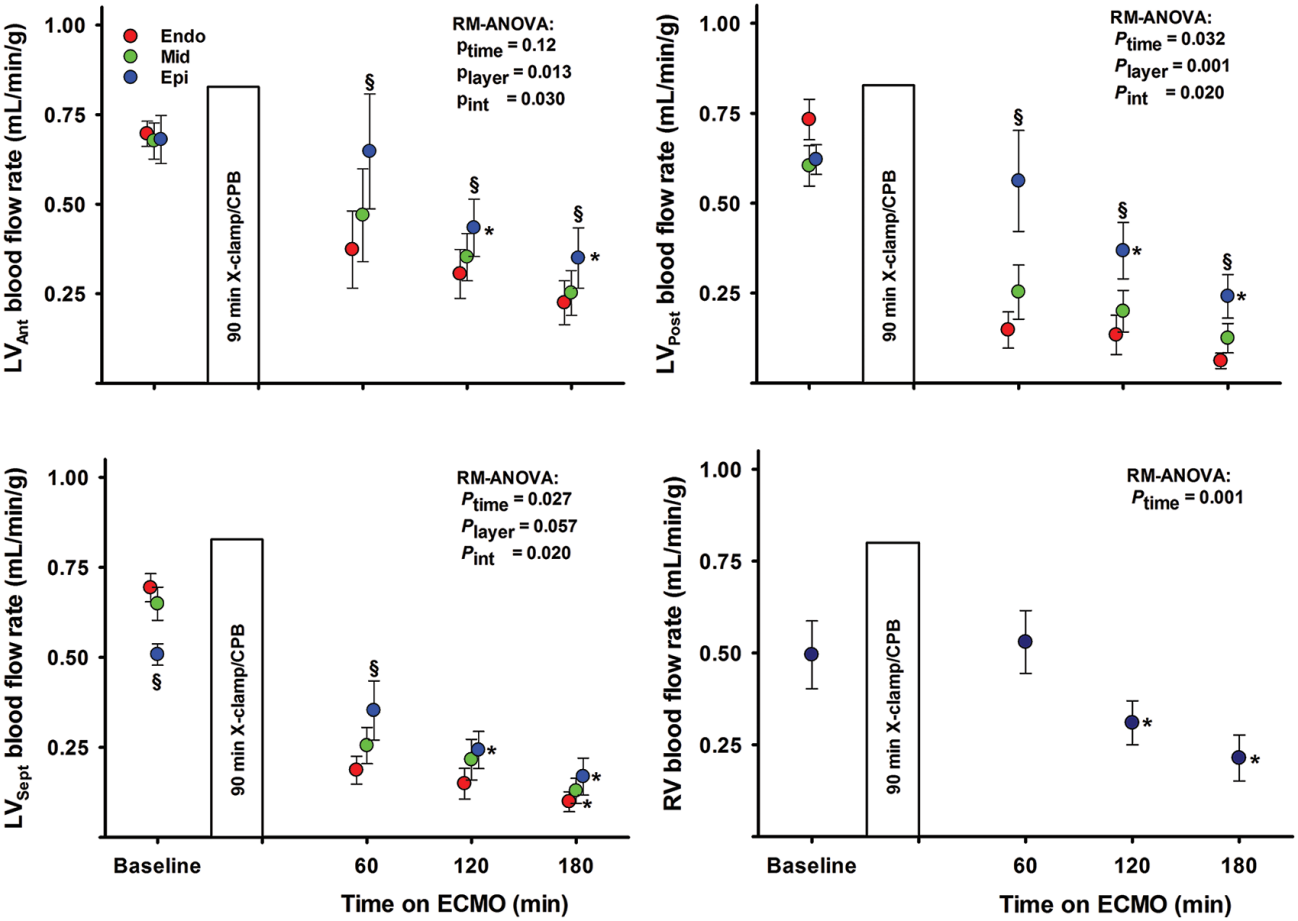
Myocardial tissue blood flow rate in the left ventricle (LV) and right ventricle (RV). Values are mean ± SEM (n = 10). Endo, Mid and Epi: subendo-, midmyo- and subepicardial wall layers; Ant, Post, Sept: anterior, posterior and septal left ventricular wall; Ptime, Player, Pint: probabilities for time, wall layer and interaction effect by repeated measurement ANOVA. *Significantly different from 60-minute ECMO within same wall layer; ^§^Significantly different from Endo at the corresponding point in time.

Myocardial tissue perfusion measured with microspheres was low in the subendocardium already after 60 minutes on ECMO-supported circulation. In the mid-and subepicardium of the LV and in the right ventricular myocardium tissue, perfusion was also reduced with time (Figure 4).

### Echocardiography

**Figure 5.**
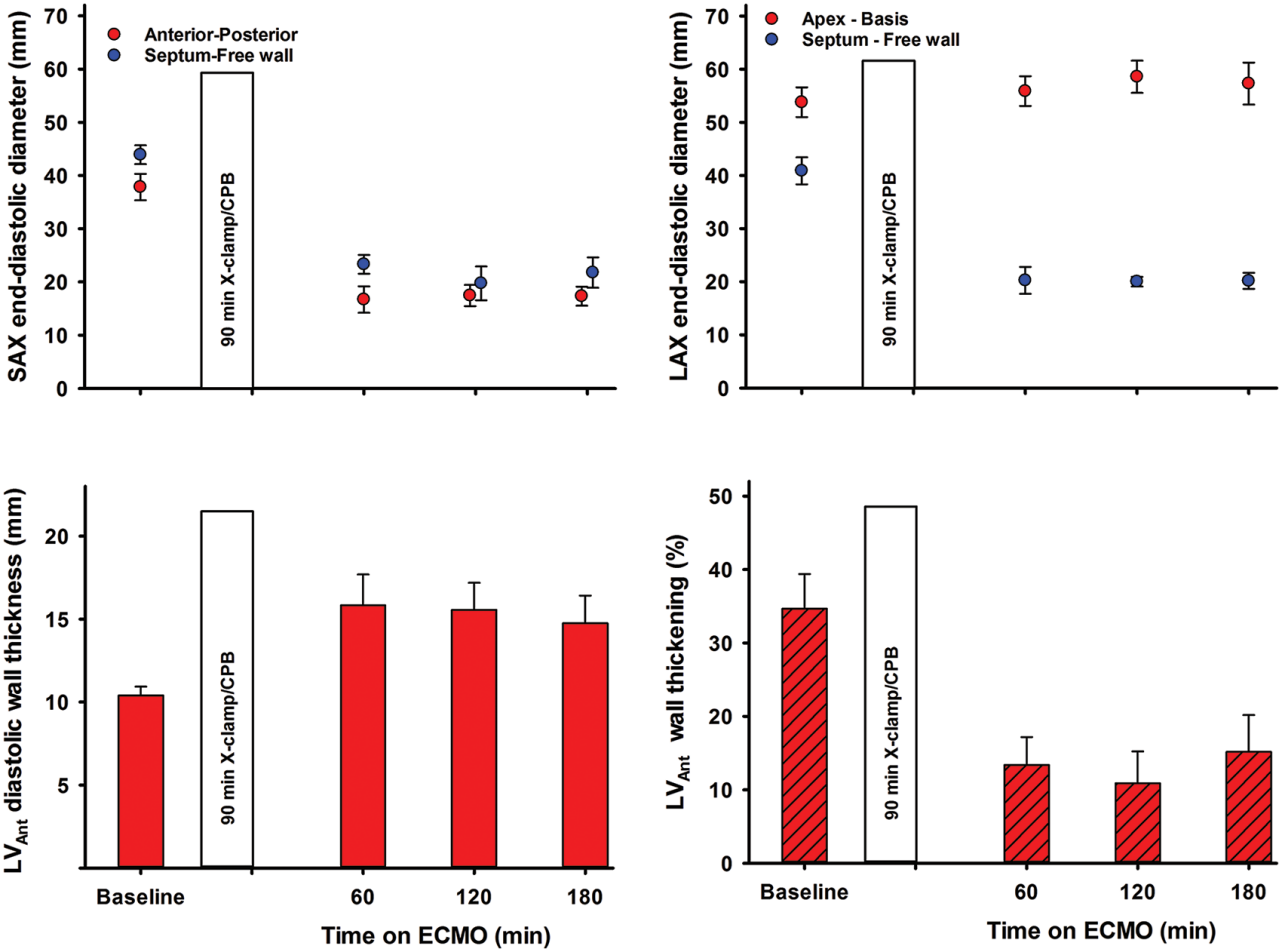
Left ventricular end-diastolic dimensions in the short-axis (SAX) and four-chamber long-axis (LAX) views and wall thickness and thickening in the left ventricular anterior wall (LVAnt). Values are mean ± SEM, bars are mean + SEM, (n = 9).

In the short-axis view, LV antero-posterior and septum to free-wall end-diastolic dimensions were reduced and unaltered during ECMO perfusion. In the long-axis view, end-diastolic septum to free-wall dimension decreased whereas the apex to basis dimension was unchanged (Figure 5). The LV remained almost empty in all animals, with only a very small ventricular cavity visible. Myocardial tissue oedema in the LV developed within the first hour after aortic declamping, demonstrated as an increase in left ventricular wall thickness. In the anterior wall in short-axis view, the left ventricular wall thickening was severely reduced during ECMO-supported perfusion. An alternating positive/negative low-velocity flow profile was frequently observed across a partly open aortic valve in most animals; left ventricular output remained negligible.

## Discussion

This experimental pig model can be used to study coronary blood flow and myocardial perfusion in a non-working heart during post-cardiotomy VA-ECMO-supported circulation. After declamping, 10 consecutive pigs with CPB and 90 minutes of aortic clamping with suboptimal cardioplegic arrest regained spontaneous cardiac rhythm after defibrillation. In pilot experiments with both 40 and 60 minutes of cross-clamp time, cardiac function was partially restored in the acute phase, and the indication for immediate ECMO-supported circulation was less clear. This experimental model thus represents an acute situation where a patient cannot be weaned from CPB after surgery.

In the acute phase, cardiac function was not restored. VA-ECMO-supported circulation was established resulting in a nearly non-pulsatile aortic pressure curve for up to 3 hours (Figure 3). In a clinical setting, a non-pulsatile aortic pressure profile during ECMO-supported circulation would prompt suspicion of pressure build-up and distension of the LV leading to a compromised coronary blood flow.^10^ Echocardiography can be used to exclude or confirm distension of the LV, and to verify whether the aortic valve opens or not; a finding usually interpreted as a sign that the LV is ejecting blood into the ascending aorta.^11^

In the present study, the flow in the ECMO circuit is set at 72 mL/min/kg, 80% of calculated full CPB flow in this pig model, a typical initial setting in a clinical situation when total circulatory support with ECMO is needed.^12^ With this setting, no haemodynamic signs of cardiac distension were observed, neither in the right nor the left side of the heart. This is in contrast to findings in a pig model without post-cardiotomy cardiogenic shock, where both LV end-systolic and end-diastolic volumes increase during ECMO-supported circulation.^13^ In the present study, the LVEDP gradually decreased from 21.0 ± 2.3 to 12.2 ± 1.5 mmHg from 15 to 180 minutes of mechanical circulatory support (Figure 3). There were no signs of left ventricular distension judged by echocardiography during the 180 minutes of supported perfusion (Figure 5). Only a minor increase in CVP_mean_ from 7.9 ± 1.1 mmHg after 15 minutes to 9.5 ± 1.2 mmHg at 180 minutes of ECMO-supported circulation was observed (Figure 2). Correspondingly, PAP_mean_ decreased from 21.7 ± 2.3 to 17.8 ± 1.3 mmHg.

In addition to the standard postoperative setup with pulse-oximetry, ECG, central venous and arterial pressures, the animals in the present study were instrumented with a left ventricular pressure catheter and a coronary artery flow probe. During the 180 minutes of VA-ECMO-supported circulation, the LVSP_max_ was close to the mean aortic blood pressure resulting in an almost flat aortic pressure curve (Figure 3). The cyclic changes in the left ventricular pressure with diastolic pressures clearly below the aortic pressure demonstrate a patent aortic valve, and an isovolumic contraction/relaxation of the LV demonstrated as LV anterior wall thickening (Figure 5). The cyclic pressure alterations in the failing LV therefore do not compromise the LAD blood flow, demonstrating flow rates at levels above or close to baseline and with high diastolic and low systolic blood flow rate during the 3-hour ECMO-support (Figure 3). The increased LAD flow after removal of the aortic cross-clamp was interpreted as post-ischaemic hyperaemia, most pronounced early after declamping. It should be noted that during a clinical ECMO-run, a situation with still-standing blood in the pulmonary circulation and the chambers of the left side of the heart over several days would induce a substantial risk of thrombus formation in the left atrium and the LV.^14^

After 60 minutes of reperfusion with ECMO-supported circulation, the myocardial perfusion measured by microspheres was reduced in the midmyocardial and the subendocardial compared to the subepicardial wall layer both in the anterior, posterior and septal regions of the LV (Figure 4). In the subepicardium and also in the right ventricular free wall, tissue perfusion decreased with time on ECMO. Myocardial tissue oedema develops within the first 15 minutes of ECMO-supported circulation, demonstrated as a 50% increase in the anterior left ventricular end-diastolic wall thickness (Figure 5).^15^ At the end of the experiments, left ventricular myocardial tissue water content averaged 84.2 ± 0.2%; clearly increased compared to 80.0 ± 0.4% found in non-ischaemic pig hearts.^16^ The development of tissue oedema might compromise myocardial perfusion.

An alternative explanation to the apparent deterioration of myocardial perfusion is the minor increase in pulsatile aortic pressure during the 180 minutes of circulatory support with ECMO (Figure 3). This could result in a watershed phenomenon in the aortic root. Arterial blood with microspheres originating from the aortic ECMO-cannula could be diluted by blood from the LV,^17–19^ and thus underestimate myocardial perfusion as computed from the microspheres. However, only occasionally, a low-velocity forward flow profile was observed with Doppler.

### Limitations

Studies of the pathophysiology in healthy hearts in an animal model limit the direct clinical relevance. Furthermore, the duration of VA-ECMO in the present study was only 3 hours, and must be extended when focusing primarily on restoration of cardiac function. Failure to decompress a severely dysfunctional LV during VA-ECMO is frequently reported to cause pulmonary oedema, a complication that may develop in the present model if the time on ECMO-supported circulation is extended.^20^

In humans, the bronchial arterioles form an anastomotic network with the pulmonary arterioles. Drainage of blood into the left atrium via the pulmonary veins contributes to volume and pressure build-up in the left atrium and ventricle. It is not clear whether this network is found in the domestic pig.^21^ However, Thebesian veins contribute to volume build-up in the left side of the heart.^22^

We observed a lack of correlation between LAD flow measured by transit-time flowmetry and myocardial perfusion measured by microspheres. We have attributed this to regional differences in the myocardium, but there is also the possibility that it could be caused by arteriovenous shunting in the myocardium.^23^

### Clinical implications

In the clinical setting, active decompression of the LV with an Impella device or a LV vent should be considered whenever the ‘breakthrough waves’ on the arterial pressure curve are less than 10 or 15 mmHg in height. This study suggests that pressure build-up and distension of the LV does not always occur in a severely affected LV with only ripples on the arterial pressure curve, and that venting of the LV during ECMO may not be needed, at least in the very early phase of post-cardiotomy circulatory support. However, left ventricular decompression should be considered when there are signs of a pressure build-up in the left atrium and ventricle.

## Conclusion

With ECMO-support and critical left ventricular failure after CPB and cardioplegic arrest, coronary artery blood flow is maintained in the acute phase, even in the presence of a non-pulsatile aortic pressure profile. However, tissue oedema rapidly develops, and myocardial perfusion decreases, earlier and more pronounced in inner wall layers of the LV.

## Supplemental Material

907557__Supplemental_file – Supplemental material for Myocardial perfusion and cardiac dimensions during extracorporeal membrane oxygenation–supported circulation in a porcine model of critical post-cardiotomy failure Click here for additional data file. Supplemental material, 907557__Supplemental_file for Myocardial perfusion and cardiac dimensions during extracorporeal membrane oxygenation–supported circulation in a porcine model of critical post-cardiotomy failure by Atle Solholm, Pirjo-Riitta Salminen, Lodve Stangeland, Christian Arvei Moen, Arve Mongstad, Bård Svenheim, Rune Haaverstad and Ketil Grong in Perfusion
