# Supplementary material for: Myocardial perfusion and cardiac dimensions during extracorporeal membrane oxygenation–supported circulation in a porcine model of critical post-cardiotomy failure
Source: Perfusion. 2020 Mar 4;35(8):763–71. doi: 10.1177/0267659120907557 (PMC7686924; doi:10.1177/0267659120907557)
Supplement: 907557__Supplemental_file – Supplemental material for Myocardial perfusion and cardiac dimensions during extracorporeal membrane oxygenation–supported circulation in a porcine model of critical post-cardiotomy failure [file 907557__Supplemental_file.pdf]

Supplemental Table A.

Arterial blood gases at Baseline and after 45 min and 85 min of aortic X-clamp. Values are mean ± SEM or median (1st quartile; 3rd quartile).

**Variable**	**Baseline**	**X-clamp 45’**	**X-clamp 85’ min**
pH	7.52 ± 0.01	7.44 ± 0.02	7.45 ± 0.02
pCO_2 (kPa)	5.0 ± 0.1	6.0 ± 0.3	5.8 ± 0.3
pO_2 (kPa)	26.6 ± 1.0	21.2 (17.6; 27.5)	18.3 (14.6; 19.5)
BE (mmol/L)	6.5 ± 0.4	4.8 ± 0.7	4.8 ± 0.4
HCO_3^- (mmol/L)	29.8 ± 0.4	29.5 ± 0.6	29.3 ± 0.5
Na^+ (mmol/L)	141 ± 1	142 ± 1	141 ± 1
K^+ (mmol/L)	3.5 ± 0.1	3.6 ± 0.1	3.6 ± 0.1
Cl^- (mmol/L)	103 ± 1	104 (102; 105)	104 (103; 104)
Hb (g/dL)	8.8 ± 0.3	8.8 ± 0.4	8.8 ± 0.4
Hct (%)	26.4 ± 0.8	26.4 ± 1.0	26.4 ± 1.1
S_aO_2 (%)	99 ± 1	98 ± 1	97 ± 1

S_aO_2 = capillary oxygen saturation by pulse oximetry.

Supplemental Table B

Rectal temperature and arterial blood gases at 60, 120 and 180 min on ECMO. Values are mean ± SEM or median (1st quartile; 3rd quartile).

**Variable**	**1 h (A)**	**2 h (B)**	**3h (C)**	**ANOVA p-value**
$Temp_{rect}$ (^0C)	38.2 (38.0; 38.3)	38.3 (38.1; 38.4)	38.2 (38.0; 38.3)	0.39
pH	7.46 (7.41; 7.50)	7.45 (7.41; 7.49)	7.44 (7.42; 7.47)	0.93
pCO_2 (kPa)	5.1 (4.9; 6.0)	5.4 (4.8; 6.0)	5.6 (5.3; 6.0)	0.91
pO_2 (kPa)	23.9 ± 4.6	23.4 ± 5.0	23.4 ± 5.2	0.94
BE (mmol/L)	3.2 (2.6; 3.8)	3.1 (1.8; 4.3)	3.8 (2.3; 4.5)	0.67
HCO_3^- (mmol/L)	27.5 ± 0.3	27.4 ± 0.4	27.9 ± 0.4	0.43
Na^+ (mmol/L)	141 (140; 142)	141 (140; 142)	141 (140; 141)	0.50
K^+ (mmol/L)	3.9 ± 0.1	4.4 ± 0.2[a,c]	4.8 ± 0.2[a,b]	< 0.001
Cl^- (mmol/L)	105 (104; 106)	104 (102; 105)	105 (103; 105)	0.53
Hb (g/dL)	8.3 ± 0.4	8.2 ± 0.4	7.7 ± 0.4	0.11
Hct (%)	25.0 ± 1.2	24.8 ± 1.3	23.3 ± 0.2	0.090
S_aO_2 (%)	97 (95; 100)	98 (94; 100)	97 (94; 99)	0.69

ANOVA = analysis of variance for repeated measurements or by Friedman Repeated Measures Analysis of Variance on Ranks. [a, b, c] = denotes significant difference from value(s) in column marked with corresponding capital letters.
